# Fluid balance-adjusted creatinine at initiation of continuous venovenous hemofiltration and mortality. A post-hoc analysis of a multicenter randomized controlled trial.

**DOI:** 10.1371/journal.pone.0197301

**Published:** 2018-06-06

**Authors:** Susanne Stads, Louise Schilder, S. Azam Nurmohamed, Frank H. Bosch, Ilse M. Purmer, Sylvia S. den Boer, Cynthia G. Kleppe, Marc G. Vervloet, Albertus Beishuizen, Armand R. J. Girbes, Pieter M. ter Wee, Diederik Gommers, A. B. Johan Groeneveld, Heleen M. Oudemans-van Straaten, for the CASH study group

**Affiliations:** 1 Department of Intensive care, Erasmus Medical Center, Rotterdam, the Netherlands; 2 Department of Intensive care, Ikazia Hospital, Rotterdam, the Netherlands; 3 Department of Nephrology, VU University Medical Center, Amsterdam, the Netherlands; 4 Department of Intensive Care, Rijnstate Hospital, Arnhem, the Netherlands; 5 Department of Intensive care, Haga hospital, den Haag, the Netherlands; 6 Department of Intensive care, Spaarne Gasthuis, Hoofddorp, the Netherlands; 7 Department of Intensive care, Noordwest Ziekenhuis groep, Alkmaar, the Netherlands; 8 Department of Intensive care, VU University Medical Center, Amsterdam, the Netherlands; 9 Department of Intensive care, Medical Spectrum, Twente, the Netherlands; University of Milan, ITALY

## Abstract

**Introduction:**

Acute kidney injury (AKI) requiring renal replacement therapy (RRT) is associated with high mortality. The creatinine-based stage of AKI is considered when deciding to start or delay RRT. However, creatinine is not only determined by renal function (excretion), but also by dilution (fluid balance) and creatinine generation (muscle mass). The aim of this study was to explore whether fluid balance-adjusted creatinine at initiation of RRT is related to 28-day mortality independent of other markers of AKI, surrogates of muscle mass and severity of disease.

**Methods:**

We performed a post-hoc analysis on data from the multicentre CASH trial comparing citrate to heparin anticoagulation during continuous venovenous hemofiltration (CVVH). To determine whether fluid balance-adjusted creatinine was associated with 28-day mortality, we performed a logistic regression analysis adjusting for confounders of creatinine generation (age, gender, body weight), other markers of AKI (creatinine, urine output) and severity of disease.

**Results:**

Of the 139 patients, 32 patients were excluded. Of the 107 included patients, 36 died at 28 days (34%). Non-survivors were older, had higher APACHE II and inclusion SOFA scores, lower pH and bicarbonate, lower creatinine and fluid balance-adjusted creatinine at CVVH initiation. In multivariate analysis lower fluid balance-adjusted creatinine (OR 0.996, 95% CI 0.993–0.999, p = 0.019), but not unadjusted creatinine, remained associated with 28-day mortality together with bicarbonate (OR 0.869, 95% CI 0.769–0.982, P = 0.024), while the APACHE II score non-significantly contributed to the model.

**Conclusion:**

In this post-hoc analysis of a multicentre trial, low fluid balance-adjusted creatinine at CVVH initiation was associated with 28-day mortality, independent of other markers of AKI, organ failure, and surrogates of muscle mass, while unadjusted creatinine was not. More tools are needed for better understanding of the complex determinants of “AKI classification”, “CVVH initiation” and their relation with mortality, fluid balance is only one.

## Introduction

Acute kidney injury (AKI) in critically ill patients is an independent risk factor for increased morbidity and mortality. Despite improved recognition and treatment, mortality rates remain between 40 and 60% [1]. Nowadays, AKI is staged by the ratio of actual serum creatinine to pre-admission serum creatinine (Risk, Injury, Failure, Loss, End stage renal disease (RIFLE), Acute Kidney Injury Network (AKIN), Kidney Disease: Improving Global Outcomes (KDIGO)), thereby defining three stages of AKI severity [2–5]. Several studies explored the relation between creatinine-based criteria of AKI at initiation of continuous renal replacement therapy (CRRT) and mortality. Bagshaw et al. found that a lower creatinine, was associated with high mortality [6]. Recently, two randomized controlled trials evaluated the effect of creatinine-based criteria to initiate CRRT on mortality using the KDIGO stage of AKI and found controversial results: either a survival benefit for starting at a lower stage of AKI (stage 2) [7], or no difference in mortality when starting at stage 3 (early) or later when complications developed [8]. Two observational studies reported that a lower creatinine at initiation of CRRT had a poor prognosis [9, 10]. However, the use of AKI stage, as a marker for severity of AKI and initiation of RRT has several limitations. Plasma creatinine concentration, the cornerstone of AKI staging, is not only determined by renal excretion, but also by hemodilution (caused by fluid accumulation) and by creatinine generation, e.g. by muscle mass. Lower creatinine levels due to fluid overload or low creatinine generation therefore underestimate true renal function impairment in critically ill patients. In none of the above mentioned studies creatinine was adjusted for fluid-balance [6–10].

The effect of fluid balance on AKI classification and outcomes was initially evaluated in a post-hoc analysis of the Fluid and Catheter Treatment Trial [11]. The study showed that patients who had AKI after adjustment for fluid balance (but not before) had worse outcomes than patients who had no AKI before and after adjustment for fluid balance. The modulating effect of fluid overload on the diagnosis of AKI using serum creatinine was recently evaluated by Macedo et al. [12]. They concluded that dilution of serum creatinine by fluid accumulation leads to underestimation of severity of AKI and delays the identification of a 50% increase in serum creatinine in critically ill patients. They developed a formula to adjust serum creatinine for fluid accumulation.

The aim of the present explorative study was to evaluate whether fluid balance-adjusted serum creatinine at CRRT initiation is related to mortality independent of other markers of severity of AKI, surrogate markers of muscle mass (age, sex, race and body weight) and severity of disease.

## Methods

We performed a post-hoc analysis of data from a multi-center randomized controlled trial, comparing citrate and heparin anticoagulation during continuous venovenous hemofiltration (CVVH) [13]. Mortality between groups was not different. The study included patients requiring CVVH for AKI in 10 participating ICUs in the Netherlands. The study was performed in accordance with the declaration of Helsinki. The study was registered at clinicaltrials.gov number NCT00209378. The ethical committee VU medical Center approved this study. The local medical ethical committees of the participating centers approved this study. Written informed consent was obtained from all participants or their legal representative.

### Study population

Between April 2005 and March 2011, patients were prospectively screened for inclusion in the CASH trial. The study included adult patients requiring CVVH for AKI and excluded patients older than 80 years, patients with an increased bleeding risk, with a known heparin induced thrombocytopenia (HIT) and patients needing therapeutic systemic anticoagulation. Patients were randomized to receive heparin or citrate anticoagulation for CVVH in predilution mode, with predilution replacement flow rates between 2000 and 4000 ml/h, according to local guidelines. For the present study, patients were post-hoc excluded when no creatinine at initiation of CVVH was available, or when a documented diagnosis of intrinsic renal disease (such as renal artery stenosis, diabetic nephropathy, nephrotic syndrome or nephrosclerosis) was documented in the medical record. The reason to exclude these patients was that the cause of worsening renal function could have been related to the underlying renal disease and not to critical illness-related AKI. The diagnosis of AKI was made by the attending physician and the decision to initiate CVVH was based on the local protocol. Data were collected using the hospital patient data management system.

### Data collection

The following baseline data were collected: age, gender, weight and race as surrogates for muscle mass, reason for ICU admission and cause of AKI (presumed as ischemic, septic or other/toxic). At initiation of CVVH the following data were obtained: number of days at ICU before CVVH initiation, cumulative fluid balance 3 days prior to initiation, diuresis 24 hours prior to initiation, severity scores: APACHE (Acute physiology and Chronic Health Evaluation) II score at ICU admission and SOFA (Sequential Organ Failure Assessment) score at CVVH initiation, creatinine at ICU admission (μmol/L), creatinine at initiation of CVVH (μmol/L). Creatinine corrected for 3 day cumulative fluid balance was calculated according to the formula defined by Macedo et al. [12]. Adjusted creatinine = initiation creatinine x ((hospital admission weight (kg) x 0.6 + ∑ (3 day cumulative fluid balance(L)))/ (hospital admission weight x 0.6)). The KDIGO stage at initiation was calculated using only the delta creatinine criteria according to the KDIGO guidelines [2]. Unfortunately no pre-morbid creatinine was available in this post-hoc analysis. We therefore used admission creatinine as baseline creatinine. When patients were admitted with a single high creatinine and need of direct RRT, the attending physician diagnosed AKI, when there was no history of chronic kidney disease and the patient also had low urine output and other uremic symptoms. The initiation of RRT classified these patients directly to KDIGO 3 [5].

### Endpoints

The primary endpoint was mortality at 28 days after CVVH initiation.

### Statistical analysis

Variables were tested for normal distribution using the Kolmogorov-Smirnov test. Normally distributed variables are expressed as mean (standard deviation), non-normally distributed variables as median [interquartile range], and categorical data as number and percentage. Unpaired Student’s t-test, Mann-Whitney-U test, Chi-square test or Fisher exact test was used, where appropriate. Statistical significance was defined as p < 0.05.

To determine the association between fluid balance-adjusted serum creatinine and 28-day mortality, logistic regression analysis was performed using backward stepwise likelihood ratio including a maximum of n/10 variables choosing those variables that had a p<0.10 in univariate analysis as confounders [14], including fluid balance because of its known association with mortality [15, 16]. For diuresis, a z-score was calculated to obtain the OR for the change per standard deviation in logistic regression. A p-value of 0.10 was used for entry and removal.

ROC curve analysis was used to define the cut-off value of fluid balance-adjusted creatinine at CVVH initiation with best prediction for 28-day mortality in MedCalc®, version 15.6.1 using the Youden index. This cut-off value was used to plot Kaplan Meier curves comparing the time to survival between patients with low adjusted CVVH initiation creatinine to patients with high adjusted CVVH initiation creatinine. The log-rank test was used to demonstrate differences.

## Results

### Flowchart

**Fig 1:**
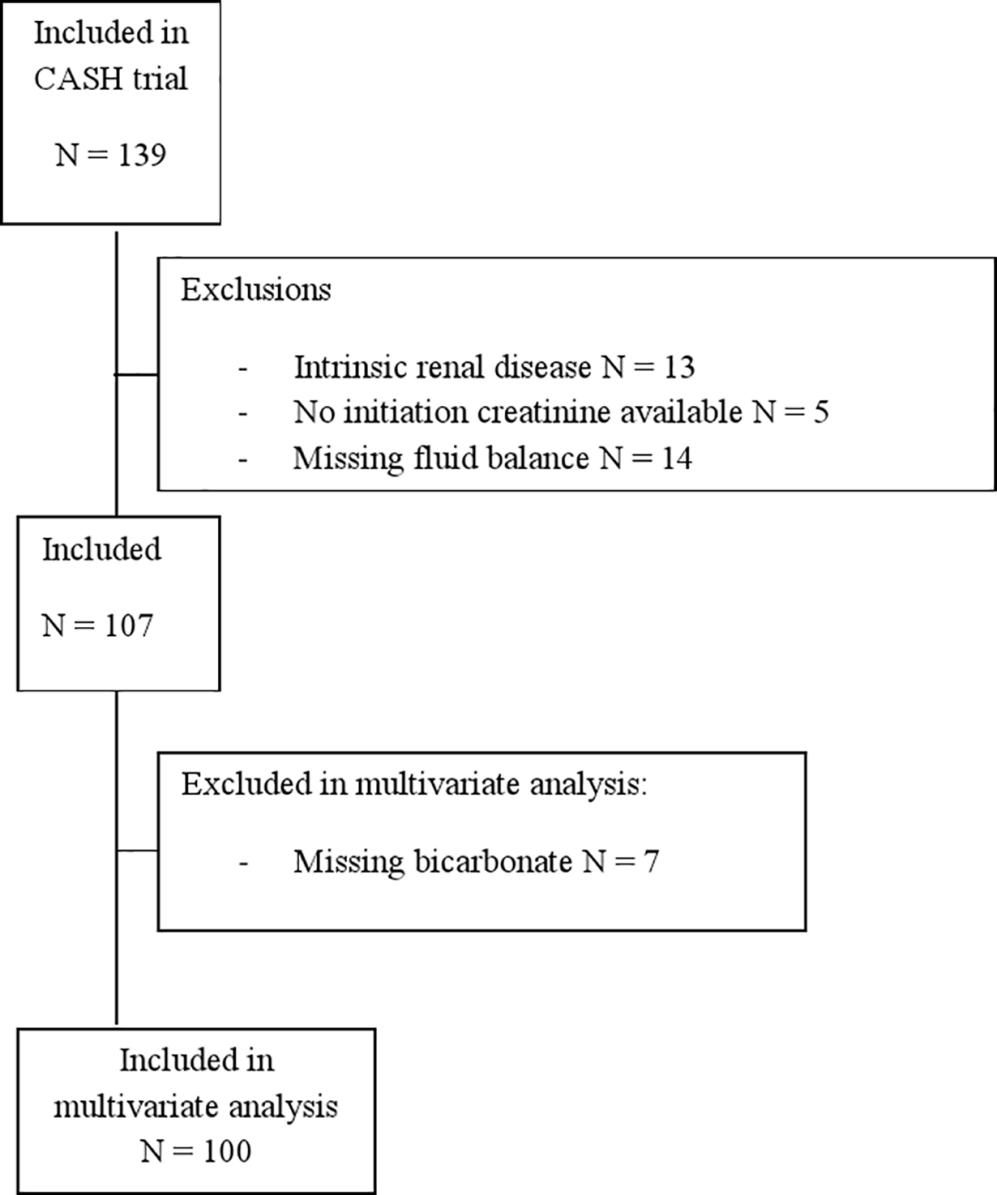
Flowchart of included and excluded patients.

Of the 139 patients included in the CASH trial, 32 patients were excluded, 13 because of a history of intrinsic renal disease, 5 patients because there was no creatinine available at the day of CVVH initiation and 14 patients because fluid balance was not available, so creatinine could not be corrected for fluid balance. In 7 patients bicarbonate was not available, these additional 7 patients were not included in the multivariate analysis (Fig 1). Altogether, 107 patients were included in the primary analysis and 100 patients in the multivariate analysis.

### Patient characteristics according to 28-day outcome

Thirty-six out of the 107 patients (34%) did not survive at day 28. Patients who died were older (72 [15] vs. 64 [15] years, p = 0.016), had higher APACHE II scores (25 (9) vs. 22 (7), p = 0.043), lower bicarbonate (17.8 (4.3) mmol/L vs. 20.4 (4.1) mmol/L, p = 0.005), lower creatinine (278 (122) μmol/L vs. 347 (155) μmol/L, p = 0.022), and a lower fluid balance-adjusted creatinine at initiation (313 (132) μmol/L vs. 388 (168) μmol/L, p = 0.022) compared to patients alive at 28 days. Urine output, KDIGO stage, fluid balance, gender, weight, admission creatinine, reason for ICU admission, days in the ICU, predilution dose and cause of AKI, were not significantly different between groups. Baseline characteristics are shown in Table 1.

**Table 1:** Baseline characteristics of cohort, according to 28-day outcome.

	Alive at 28 days, n = 71	Dead at 28 days, n = 36	P-value
Age, years	64 [15]	72 [15]	0.016
Male gender, nr (%)	50 (70)	22 (61)	0.332
Race, white, nr (%)	47 (64)	23 (64)	0.813
Weight, kg	83 [24]	86 [28]	0.789
Reason ICU admission, nr (%)			
Circulatory failure	14 (20)	9 (25)	0.636
Respiratory failure	33 (46)	16 (44)	
Trauma	3 (4)	1 (3)	
Post CPR	2 (3)	3 (8)	
Post-operative	19 (27)	7 (20)	
Cause of acute kidney injury, nr (%)			
Sepsis	31 (44)	14 (39)	0.731
Ischemic	38 (53)	20 (56)	
Other	2 (3)	2 (5)	
Creatinine admission, μmol/L	121 [110]	118 [168]	0.275
APACHE II	22 (7)	25 (9)	0.043
SOFA score	10 [5]	11 (4)	0.130
ICU admission before CVVH, days	2 [4]	3 [5]	0.594
Potassium, mmol/L	4.7 (0.8)	4.7 (0.7)	0.822
pH	7.29 (0.11)	7.25 (0.11)	0.090
Bicarbonate, mmol/L	20.4 (4.1)	17.8 (4.3)	0.005
**At start CRRT**			
Cumulative fluid balance 3 days before start, ml	5556 [6484]	7102 (6142)	0.609
Diuresis in 24 hr prior to CVVH, ml	341 [851]	410 [1043]	0.617
Creatinine start CVVH, μmol/L	347 (155)	278 (122)	0.022
Fluid balance-adjusted creatinine at start, μmol/L	388 (168)	313 (132)	0.022
Predilution dose, ml/kg/hr	22 (5)	21 (6)	0.751
KDIGO stage, nr (%):	71 (100)	36 (100)	0.541
KDIGO 1	10 (14)	6 (17)	
KDIGO 2	14 (20)	10 (28)	
KDIGO 3	47 (66)	20 (55)	

Mean (standard deviation) for normally distributed variables, median [interquartile range] for non-normally distributed variables, number (percentage) when appropriate; CPR, cardiopulmonary resuscitation; APACHE II, acute physiology and chronic health evaluation score; SOFA, sequential organ failure assessment; CVVH continuous venovenous hemofiltration, KDIGO, kidney disease: improving global outcomes.

### Relation between fluid balance-adjusted creatinine at CVVH initiation and 28-day mortality

To determine the association between fluid balance-adjusted creatinine and 28-day mortality, variables that were potentially associated with mortality were first tested in univariate logistic regression analysis. In this analysis lower bicarbonate (OR 0.853, 95% CI 0.758–0.960, p = 0.008), lower creatinine at CVVH initiation (OR 0.996, 95% CI 0.993–1.000, p = 0.026), and lower fluid balance-adjusted creatinine at initiation (OR 0.997, 95% CI 0.994–1.000, p = 0.026) were associated with mortality (Table 2). The relation with APACHE score tended to significance (OR 1.058, 95% CI 1.000–1.119, p = 0.050).

**Table 2:** Univariate logistic regression analysis of variables associated with 28 day mortality.

	OR	95% CI	p-value
Age, years	1.039	1.000–1.081	0.053
Male gender	0.660	0.284–1.532	0.333
Race, white	0.903	0.390–2.091	0.813
Weight, kg	1.010	0.994–1.026	0.210
Creatinine at start CVVH, μmol/L	0.996	0.993–1.000	0.026
Cumulative fluid balance 3 days before start CVVH	1.000	1.000–1.000	0.382
Apache II	1.058	1.000–1.112	0.050
SOFA day 0	1.113	0.986–1.256	0.084
pH	0.035	0.001–1.761	0.093
Bicarbonate, mmol/L	0.853	0.758–0.960	0.008
Diuresis (z-score)	1.121	0.727–1.728	0.605
Fluid balance-adjusted creatinine at start, μmol/L	0.997	0.994–1.000	0.026
KDIGO stage			
KDIGO 1	1		0.543
KDIGO 2	1.190	0.325–4.356	0.792
KDIGO 3	0.790	0.227–2.216	0.554

APACHE II, acute physiology and chronic health evaluation score, OR, Odds ratio, SOFA, sequential organ failure assessment, KDIGO, kidney disease: improving global outcomes. For continuous variables the odds ratios are per unit increase. For diuresis, Z-transformation was performed; this odds ratio is per standard deviation increase.

Subsequently, logistic regression was performed including both creatinine and fluid balance-adjusted creatinine, APACHE II score, bicarbonate and fluid balance known to be associated with mortality [15, 16]. APACHE score was included as marker of severity of disease and not SOFA score, because of the lower p-value of APACHE in univariate analysis. Age was not included because age is a component of the APACHE score. After covariate adjustment lower fluid balance-adjusted initiation creatinine (OR 0.996, 95% CI 0.993–0.999, p = 0.019), but not unadjusted creatinine (lost in second step, first step: OR 1.021, 95% CI 0.987–1.056, p 0.228), remained independently associated with 28-day mortality together with lower bicarbonate (OR 0.869, 95% CI 0.769–0.982, p = 0.024), while APACHE II score non-significantly contributed to the model (Table 3).

**Table 3:** Multivariate logistic regression analysis of variables associated with 28-day mortality.

	OR	95% CI	p-value
APACHE II score	1.060	0.994–1.129	0.075
Bicarbonate, mmol/L	0.869	0.769–0.982	0.024
Fluid balance-adjusted creatinine start, μmol/L	0.996	0.993–0.999	0.019

OR, Odds ratio. The odds ratios are per unit increase.

**Variables included**: unadjusted creatinine, cumulative fluid balance, APACHE II score, Bicarbonate, adjusted creatinine.

**Variables removed**: step 2: unadjusted creatinine was lost, step 3: cumulative fluid balance was lost

To determine the cut-off value of the adjusted creatinine at initiation with the best association with 28-day mortality, ROC-curve analysis was performed. In this analysis, a fluid balance-adjusted creatinine of 361 μmol/L appeared to be associated best with 28-day mortality.

**Fig 2:**
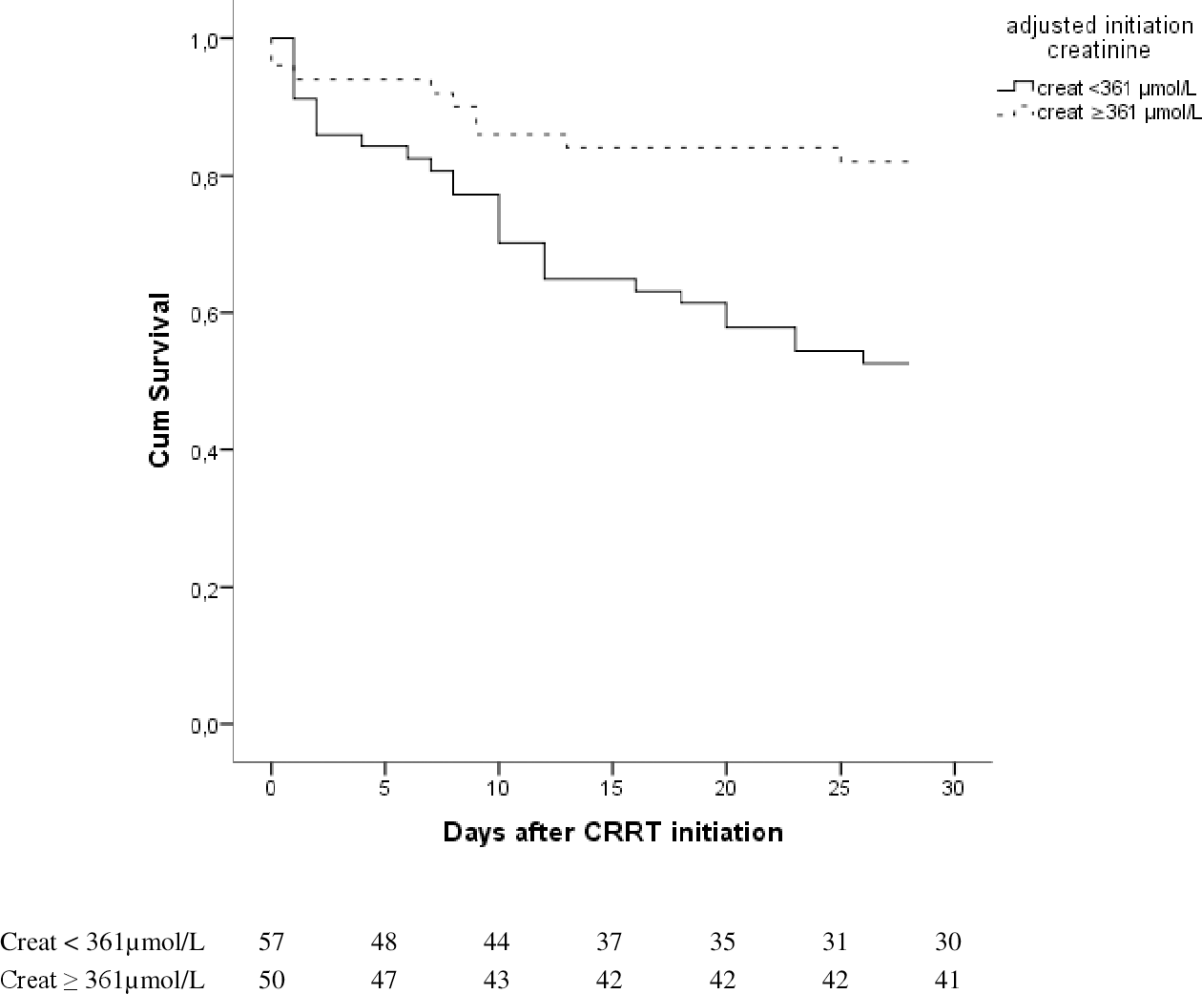
28-day survival curves according to the optimal fluid balance-adjusted creatinine at initiation of CVVH.

Kaplan Meier survival curve analysis showed a significant difference between survival curves for patients with CVVH initiation at an adjusted creatinine below 361 μmol/L and those equal to or above 361 μmol/L (log-rank p = 0.002) (Fig 2). Patients with CVVH initiation at lower fluid balance-adjusted creatinine levels than 361 μmol/L had poorer survival.

## Discussion

### Key findings

In this post-hoc analysis of the database of a prospective randomized controlled multi-center trial, we found that lower fluid balance-adjusted creatinine at initiation of CVVH was independently associated with higher 28-day mortality while unadjusted creatinine (after covariate correction) and KDIGO staging were not. This association was independent of muscle mass-related confounders of creatinine (age, body weight, race), markers of severity of AKI (bicarbonate, urine output, creatinine, KDIGO criteria) and severity of disease. The optimal cut-off value in the present population for a fluid balance-adjusted creatinine was 361 μmol/L. Mortality was higher in the patients in whom CVVH was initiated at a fluid balance-adjusted creatinine below 361 μmol/L.

The interpretation of low fluid balance-adjusted creatinine is complex because creatinine is a marker of the balance between creatinine generation (muscle mass) and creatinine excretion (renal function). Low serum creatinine can therefore be considered as a low muscle mass or as an earlier stage of AKI. The presently found relation may therefore indicate that either initiation of CRRT at an earlier stage of AKI or low muscle mass at CRRT initiation are associated with higher mortality, or both.

### The role of fluid balance

Apart from muscle mass and severity of AKI, fluid overload is an important confounder for mortality. Fluid overload is a dual confounder. Fluid overload itself is a severe complication of critical illness and independently associated with worse outcome, especially in patients with AKI [15–18]. Furthermore, fluid overload dilutes serum creatinine and thereby underestimates the severity of AKI and delays its diagnosis [12]. In a post-hoc analysis of the ARDS network trial, patients who met the criteria for AKI after correction for fluid balance (and not before) had a greater mortality than those who did not meet AKI criteria (before and after correction) and those who had AKI before but not after adjustment for fluid balance [11]. In another study, patients in whom AKI was diagnosed only after adjustment for fluid balance had higher mortality than patients without AKI [19]. To account for these dual effects of fluid balance, we both adjusted creatinine for fluid balance and added fluid balance as an independent factor in the multivariate logistic regression analysis.

### Creatinine generation

Previous studies have shown an association between reduced creatinine generation during hemodialysis [20], low serum creatinine at ICU admission (< 30 μmol/L) and low peak plasma creatinine concentrations (< 60 μmol/L) with mortality [21, 22]. That low creatinine may reflect reduced muscle mass has been demonstrated by Baxmann et al. using cystatin C as a marker of renal function [23]. To adjust for the confounding of serum creatinine by low muscle mass, we added surrogates for muscle mass in our multivariate regression analysis. Age is one of the determinants of the APACHE II score and therefore covered by adding APACHE II score in the multivariate analysis. Age has dual effects. A higher age is associated with higher mortality per se, while on the other hand muscle mass declines with aging. Body weight, gender and race were not included because we found no association with 28-day mortality in univariate analysis. We do however admit that the present correction for confounders of muscle mass is insufficient. Low body weight does not necessarily implicate low muscle mass and high body weight may be associated with low muscle mass (sarcopenic obesity). Furthermore, the relation between high age and low muscle mass is not straightforward. Thus, whether the present results suggest that low muscle mass at CVVH initiation is associated with increased mortality cannot be excluded.

### Creatinine excretion

Serum creatinine is primarily conceived as a marker of renal excretory function and the different AKI classifications are based on this concept. Remarkably, while low fluid balance-adjusted creatinine was associated with mortality, neither unadjusted creatinine nor the stage of AKI according to the KDIGO criteria was associated with mortality in this study. The determination of AKI stage was as reliable as possible because we excluded patients with missing creatinine before initiation of CVVH. As recently discussed by Chawla et al. it is important to consider the timeframe of development of kidney injury to accurately classify these patients [5]. However, because premorbid creatinine values were not available we used baseline instead of pre-admission creatinine which can be conceived as limitation. In patients with a high admission creatinine and direct need of RRT the attending physician diagnosed AKI (and not CKD), based on clinical data. These patients were staged as KDIGO 3, because of immediate initiation of RRT. Nevertheless, neither KDIGO nor the previous AKI classifications (RIFLE, AKIN) consider the confounding of fluid balance.

Even when our results would suggest that initiation of CRRT at an earlier stage of AKI is associated with higher mortality, the translation of these results to clinical practice is difficult. In the present study, timing was left to the considerations of the physician in charge and it is well known that CRRT is initiated at an earlier stage of AKI in the most severely ill patients with hemodynamic instability, severe fluid overload or severe acidosis. Low bicarbonate was an independent predictor of mortality in our study. Thus, the stage of AKI will never be the sole criterion used to decide when to initiate CRRT in daily practice, the severity of illness and renal and non-renal complications like fluid overload and acidosis are always considered. Randomized controlled trials should account for this confounding.

### Timing of CRRT using creatinine based criteria

The results of studies investigating timing of CRRT using creatinine based definitions are controversial. Two systematic reviews cautiously suggested early CRRT initiation might be associated with better survival [24, 25]. However, these reviews were mainly based on low quality heterogeneous studies. In a recently published randomized controlled trial in surgical patients, initiation of CRRT at a lower creatinine (at KDIGO stage 2) was associated with lower mortality [7]. In contrast, a multicenter randomized controlled trial including patients with AKI requiring mechanical ventilation or catecholamine infusion and without potentially AKI-related life-threatening complications, found no difference in mortality between early (KDIGO stage 3) and late initiation of RRT (when a conventional indication developed, after diagnosing KDIGO stage 3) [8]. Similarly, a multicenter randomized feasibility trial found no difference in mortality between early (within 12 hours after KDGIO stage 2) and late initiation of RRT (when a conventional indication developed, after 12 hours reaching KDIGO stage 2) either [26]. In the two latter trials, serum creatinine concentration at initiation of RRT was not different between groups, and a substantial proportion of late patients did not receive RRT because of dying or renal recovery. In contrast and in agreement with our results, two observational studies reported that a lower creatinine at initiation of CRRT was associated with higher mortality [9, 10]. Recently two meta-analysis of high quality trials analyzed the impact of early or late RRT initiation on outcome [27, 28]. After exclusion of studies reporting incomplete baseline demographic data, studies without severity of illness assessment or studies with differences between cohorts at baseline, no survival benefit for early RRT initiation was found, supporting the importance of considering severity of disease when initiating CRRT. However, none of the previous studies on timing, using creatinine as a compound of AKI stage or as a solitary value, was adjusted for fluid balance. In the present study, we corrected for disease severity and baseline characteristics, as well as for the non-renal confounders of creatinine, for other markers of timing and for severity of disease, suggesting that low fluid balance-adjusted creatinine could partially be interpreted as a marker of early timing of CRRT, and that, if this were the case, early timing in this population was associated with mortality.

In contrast to previous studies, urinary output [21, 29] and days in ICU [6] were not related to mortality in our population. Urinary output may be confounded by the use of diuretics and oliguria does not necessarily implicate the presence of AKI [30].

### Strengths and limitations

Important limitations of our study are the small sample size, limiting its statistical power. Furthermore, the initiation of CVVH was not protocollized and was therefore biased. CRRT might have been started earlier in the sicker patients explaining the higher mortality. Moreover, this study was not designed to evaluate fluid balance-adjusted creatinine. Patients who needed systemic anticoagulation or had an otherwise increased risk of bleeding were excluded in the CASH trial. As a result, we included less surgical patients and less patients with septic AKI limiting the generalizability of our results. The database could, however, be used because mortality between randomized groups was not different [13]. Unfortunately we had no data on fluid balance more than three days prior to CRRT initiation. However, median stay in the ICU was 2 days, thus for the majority of patients fluid balance from admission was available. Furthermore, fluid balance may not precisely estimate fluid status, because part of the fluids may be lost by perspiration or wounds. Also we did not have an independent measure of muscle mass and had no data on premorbid creatinine. Finally, due to missing values, 7 patients were excluded in the multivariate analysis. Nevertheless, our cohort is comparable to other studies regarding disease severity, indicated by SOFA and APACHE II scores, age, vasopressor dependency and proportion of mechanically ventilated patients [6, 31, 32]. Altogether, the present study can only signal the pitfalls related to the interpretation of serum creatinine being more than a marker of renal function [33].

Our study has several strengths. The use of fluid balance-adjusted creatinine to stage AKI is unique in the available literature, and strongly recommended since recent studies showed underestimation and misclassification of AKI if uncorrected creatinine is used [11, 12, 19]. Confounding is further minimized, because we adjusted creatinine for surrogate markers of muscle mass, such as age, body weight and race. Despite these adjustments, the relation between low creatinine and mortality as shown in our study insufficiently differentiates between an earlier initiation of CVVH, a low muscle mass or both as risk factors for dying in this population.

## Conclusions

In conclusion, in this post-hoc analysis of a multicenter study we found that a low fluid balance-adjusted creatinine at initiation of CVVH was associated with increased 28-day mortality independent of surrogates of muscle mass and severity of organ failure, while unadjusted creatinine and KDIGO stage were not. Because we only used surrogates for muscle mass and fluid status, the present study insufficiently differentiates whether a lower muscle mass or earlier initiation of CVVH or both are associated with mortality. Our results cannot be translated to clinical practice, but are hypothesis generating. They suggest that future studies on determinants of mortality should take fluid balance into account when investigating AKI stage as a criterion for timing of CRRT, include better markers of muscle mass such as bioimpedance analysis, and account for severity of disease and acidosis.

## Supporting information

S1 Dataset (SAV)

## References

[1] Uchino S, Kellum JA, Bellomo R, Doig GS, Morimatsu H, Morgera S, et al Acute renal failure in critically ill patients: a multinational, multicenter study. JAMA. 2005;294(7):813–8. doi: 10.1001/jama.294.7.813 16106006 10.1001/jama.294.7.813

[2] Kellum JA, Lameire N, Group KAGW. Diagnosis, evaluation, and management of acute kidney injury: a KDIGO summary (Part 1). Critical care. 2013;17(1):204 doi: 10.1186/cc11454 23394211 10.1186/cc11454 PMC4057151

[3] Mehta RL, Kellum JA, Shah SV, Molitoris BA, Ronco C, Warnock DG, et al Acute Kidney Injury Network: report of an initiative to improve outcomes in acute kidney injury. Critical care. 2007;11(2):R31 doi: 10.1186/cc5713 17331245 10.1186/cc5713 PMC2206446

[4] Bellomo R, Ronco C, Kellum JA, Mehta RL, Palevsky P, Acute Dialysis Quality Initiative w. Acute renal failure—definition, outcome measures, animal models, fluid therapy and information technology needs: the Second International Consensus Conference of the Acute Dialysis Quality Initiative (ADQI) Group. Critical care. 2004;8(4):R204–12. doi: 10.1186/cc2872 15312219 10.1186/cc2872 PMC522841

[5] Chawla LS, Bellomo R, Bihorac A, Goldstein SL, Siew ED, Bagshaw SM, et al Acute kidney disease and renal recovery: consensus report of the Acute Disease Quality Initiative (ADQI) 16 Workgroup. Nature reviews Nephrology. 2017;13(4):241–57. doi: 10.1038/nrneph.2017.2 28239173 10.1038/nrneph.2017.2

[6] Bagshaw SM, Uchino S, Bellomo R, Morimatsu H, Morgera S, Schetz M, et al Timing of renal replacement therapy and clinical outcomes in critically ill patients with severe acute kidney injury. Journal of critical care. 2009;24(1):129–40. doi: 10.1016/j.jcrc.2007.12.017 19272549 10.1016/j.jcrc.2007.12.017

[7] Zarbock A, Kellum JA, Schmidt C, Van Aken H, Wempe C, Pavenstadt H, et al Effect of Early vs Delayed Initiation of Renal Replacement Therapy on Mortality in Critically Ill Patients With Acute Kidney Injury: The ELAIN Randomized Clinical Trial. JAMA. 2016;315(20):2190–9. doi: 10.1001/jama.2016.5828 27209269 10.1001/jama.2016.5828

[8] Gaudry S, Hajage D, Schortgen F, Martin-Lefevre L, Pons B, Boulet E, et al Initiation Strategies for Renal-Replacement Therapy in the Intensive Care Unit. The New England journal of medicine. 2016;375(2):122–33. doi: 10.1056/NEJMoa1603017 27181456 10.1056/NEJMoa1603017

[9] Bagshaw SM, Wald R, Barton J, Burns KE, Friedrich JO, House AA, et al Clinical factors associated with initiation of renal replacement therapy in critically ill patients with acute kidney injury-a prospective multicenter observational study. Journal of critical care. 2012;27(3):268–75. doi: 10.1016/j.jcrc.2011.06.003 21798709 10.1016/j.jcrc.2011.06.003

[10] Ostermann M, Chang RW. Correlation between parameters at initiation of renal replacement therapy and outcome in patients with acute kidney injury. Critical care. 2009;13(6):R175 doi: 10.1186/cc8154 19889205 10.1186/cc8154 PMC2811955

[11] Liu KD, Thompson BT, Ancukiewicz M, Steingrub JS, Douglas IS, Matthay MA, et al Acute kidney injury in patients with acute lung injury: impact of fluid accumulation on classification of acute kidney injury and associated outcomes. Critical care medicine. 2011;39(12):2665–71. doi: 10.1097/CCM.0b013e318228234b 21785346 10.1097/CCM.0b013e318228234b PMC3220741

[12] Macedo E, Bouchard J, Soroko SH, Chertow GM, Himmelfarb J, Ikizler TA, et al Fluid accumulation, recognition and staging of acute kidney injury in critically-ill patients. Critical care. 2010;14(3):R82 doi: 10.1186/cc9004 20459609 10.1186/cc9004 PMC2911707

[13] Schilder L, Nurmohamed SA, Bosch FH, Purmer IM, den Boer SS, Kleppe CG, et al Citrate anticoagulation versus systemic heparinisation in continuous venovenous hemofiltration in critically ill patients with acute kidney injury: a multi-center randomized clinical trial. Critical care. 2014;18(4):472 doi: 10.1186/s13054-014-0472-6 25128022 10.1186/s13054-014-0472-6 PMC4161888

[14] Altman DG. Practical statistics for medical research. 1 ed: Chapman & Hall; 1991 p. 349.

[15] Payen D, de Pont AC, Sakr Y, Spies C, Reinhart K, Vincent JL, et al A positive fluid balance is associated with a worse outcome in patients with acute renal failure. Crit Care. 2008;12(3):R74 doi: 10.1186/cc6916 18533029 10.1186/cc6916 PMC2481469

[16] Bouchard J, Soroko SB, Chertow GM, Himmelfarb J, Ikizler TA, Paganini EP, et al Fluid accumulation, survival and recovery of kidney function in critically ill patients with acute kidney injury. Kidney Int. 2009;76(4):422–7. doi: 10.1038/ki.2009.159 19436332 10.1038/ki.2009.159

[17] Vaara ST, Korhonen AM, Kaukonen KM, Nisula S, Inkinen O, Hoppu S, et al Fluid overload is associated with an increased risk for 90-day mortality in critically ill patients with renal replacement therapy: data from the prospective FINNAKI study. Critical care. 2012;16(5):R197 doi: 10.1186/cc11682 23075459 10.1186/cc11682 PMC3682299

[18] Neyra JA, Li X, Canepa-Escaro F, Adams-Huet B, Toto RD, Yee J, et al Cumulative Fluid Balance and Mortality in Septic Patients With or Without Acute Kidney Injury and Chronic Kidney Disease. Critical care medicine. 2016;44(10):1891–900. doi: 10.1097/CCM.0000000000001835 27352125 10.1097/CCM.0000000000001835 PMC5505731

[19] Moore E, Tobin A, Reid D, Santamaria J, Paul E, Bellomo R. The Impact of Fluid Balance on the Detection, Classification and Outcome of Acute Kidney Injury After Cardiac Surgery. J Cardiothorac Vasc Anesth. 2015;29(5):1229–35. doi: 10.1053/j.jvca.2015.02.004 26005020 10.1053/j.jvca.2015.02.004

[20] Wilson FP, Sheehan JM, Mariani LH, Berns JS. Creatinine generation is reduced in patients requiring continuous venovenous hemodialysis and independently predicts mortality. Nephrology, dialysis, transplantation: official publication of the European Dialysis and Transplant Association—European Renal Association. 2012;27(11):4088–94. 10.1093/ndt/gfr809 PMC3529547 22273668

[21] Harris SK, Lewington AJ, Harrison DA, Rowan KM. Relationship between patients' outcomes and the changes in serum creatinine and urine output and RIFLE classification in a large critical care cohort database. Kidney international. 2015;88(2):369–77. doi: 10.1038/ki.2015.70 25760320 10.1038/ki.2015.70

[22] Udy AA, Scheinkestel C, Pilcher D, Bailey M, Australian, New Zealand Intensive Care Society Centre for O, et al. The Association Between Low Admission Peak Plasma Creatinine Concentration and In-Hospital Mortality in Patients Admitted to Intensive Care in Australia and New Zealand. Critical care medicine. 2016;44(1):73–82. 10.1097/CCM.0000000000001348 26474114

[23] Baxmann AC, Ahmed MS, Marques NC, Menon VB, Pereira AB, Kirsztajn GM, et al Influence of muscle mass and physical activity on serum and urinary creatinine and serum cystatin C. Clin J Am Soc Nephrol. 2008;3(2):348–54. doi: 10.2215/CJN.02870707 18235143 10.2215/CJN.02870707 PMC2390952

[24] Karvellas CJ, Farhat MR, Sajjad I, Mogensen SS, Leung AA, Wald R, et al A comparison of early versus late initiation of renal replacement therapy in critically ill patients with acute kidney injury: a systematic review and meta-analysis. Crit Care. 2011;15(1):R72 doi: 10.1186/cc10061 21352532 10.1186/cc10061 PMC3222005

[25] Seabra VF, Balk EM, Liangos O, Sosa MA, Cendoroglo M, Jaber BL. Timing of renal replacement therapy initiation in acute renal failure: a meta-analysis. Am J Kidney Dis. 2008;52(2):272–84. doi: 10.1053/j.ajkd.2008.02.371 18562058 10.1053/j.ajkd.2008.02.371

[26] Wald R, Adhikari NK, Smith OM, Weir MA, Pope K, Cohen A, et al Comparison of standard and accelerated initiation of renal replacement therapy in acute kidney injury. Kidney international. 2015;88(4):897–904. doi: 10.1038/ki.2015.184 26154928 10.1038/ki.2015.184

[27] Wierstra BT, Kadri S, Alomar S, Burbano X, Barrisford GW, Kao RL. The impact of "early" versus "late" initiation of renal replacement therapy in critical care patients with acute kidney injury: a systematic review and evidence synthesis. Crit Care. 2016;20(1):122 doi: 10.1186/s13054-016-1291-8 27149861 10.1186/s13054-016-1291-8 PMC4858821

[28] Feng YM, Yang Y, Han XL, Zhang F, Wan D, Guo R. The effect of early versus late initiation of renal replacement therapy in patients with acute kidney injury: A meta-analysis with trial sequential analysis of randomized controlled trials. PloS one. 2017;12(3):e0174158 doi: 10.1371/journal.pone.0174158 28329026 10.1371/journal.pone.0174158 PMC5362192

[29] Vaara ST, Parviainen I, Pettila V, Nisula S, Inkinen O, Uusaro A, et al Association of oliguria with the development of acute kidney injury in the critically ill. Kidney Int. 2016;89(1):200–8. doi: 10.1016/j.kint.2015.12.007 26352301 10.1038/ki.2015.269

[30] Prowle JR, Liu YL, Licari E, Bagshaw SM, Egi M, Haase M, et al Oliguria as predictive biomarker of acute kidney injury in critically ill patients. Crit Care. 2011;15(4):R172 doi: 10.1186/cc10318 21771324 10.1186/cc10318 PMC3387614

[31] Vaara ST, Reinikainen M, Wald R, Bagshaw SM, Pettila V, Group FS. Timing of RRT based on the presence of conventional indications. Clinical journal of the American Society of Nephrology: CJASN. 2014;9(9):1577–85. doi: 10.2215/CJN.12691213 25107952 10.2215/CJN.12691213 PMC4152821

[32] Shiao CC, Ko WJ, Wu VC, Huang TM, Lai CF, Lin YF, et al U-curve association between timing of renal replacement therapy initiation and in-hospital mortality in postoperative acute kidney injury. PloS one. 2012;7(8):e42952 doi: 10.1371/journal.pone.0042952 22952623 10.1371/journal.pone.0042952 PMC3429468

[33] Ostermann M, Kashani K, Forni LG. The two sides of creatinine: both as bad as each other? J Thorac Dis. 2016;8(7):E628–30. doi: 10.21037/jtd.2016.05.36 27501529 10.21037/jtd.2016.05.36 PMC4958791

